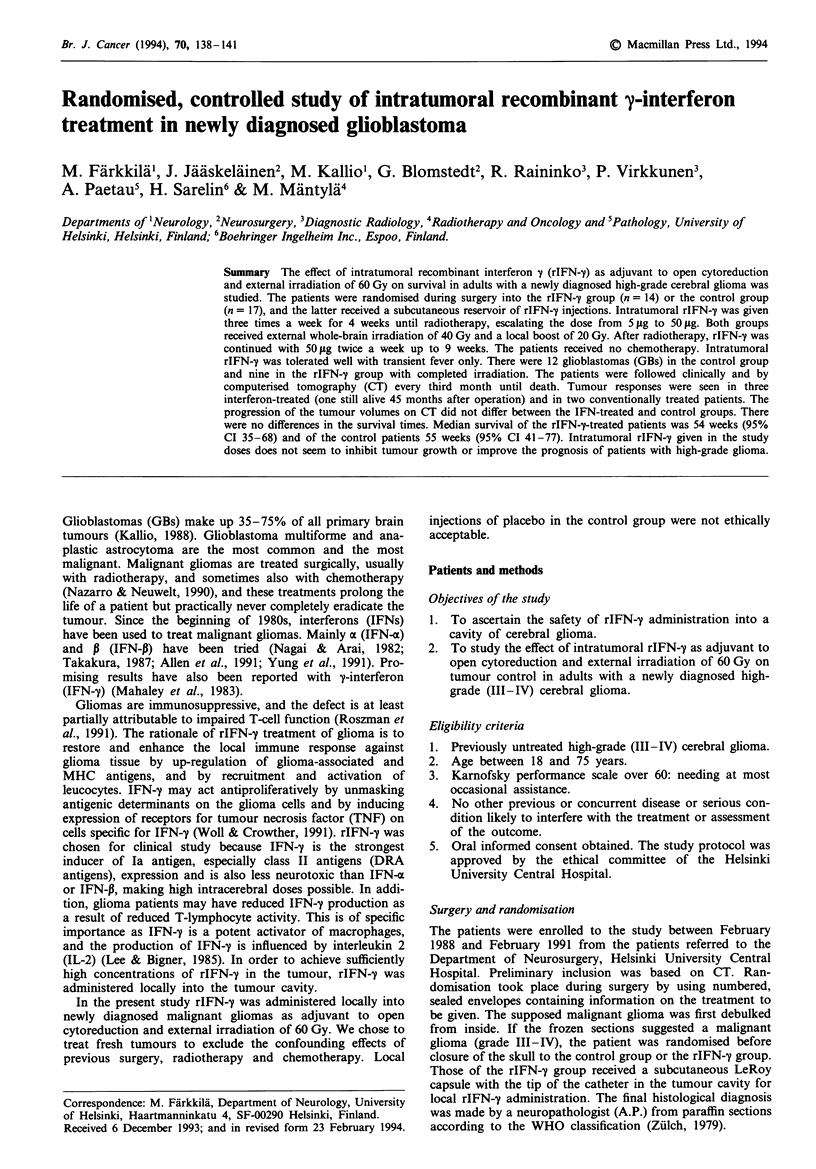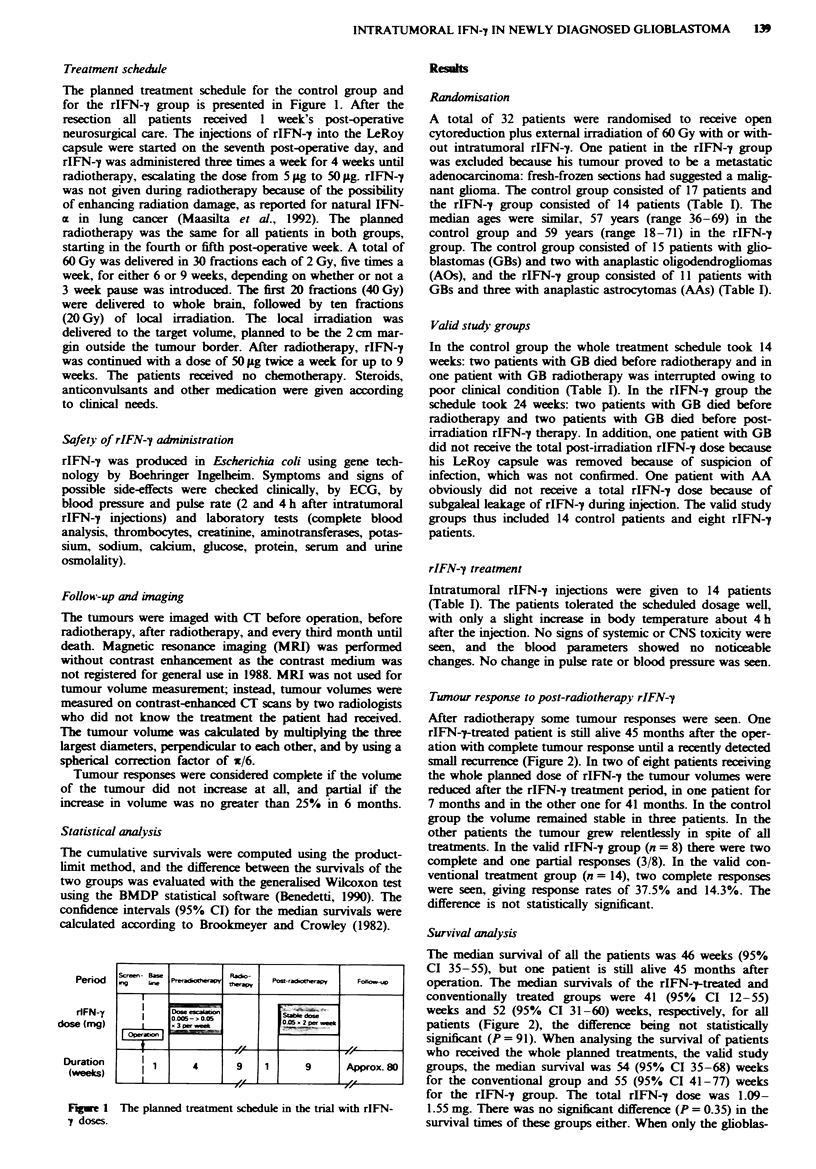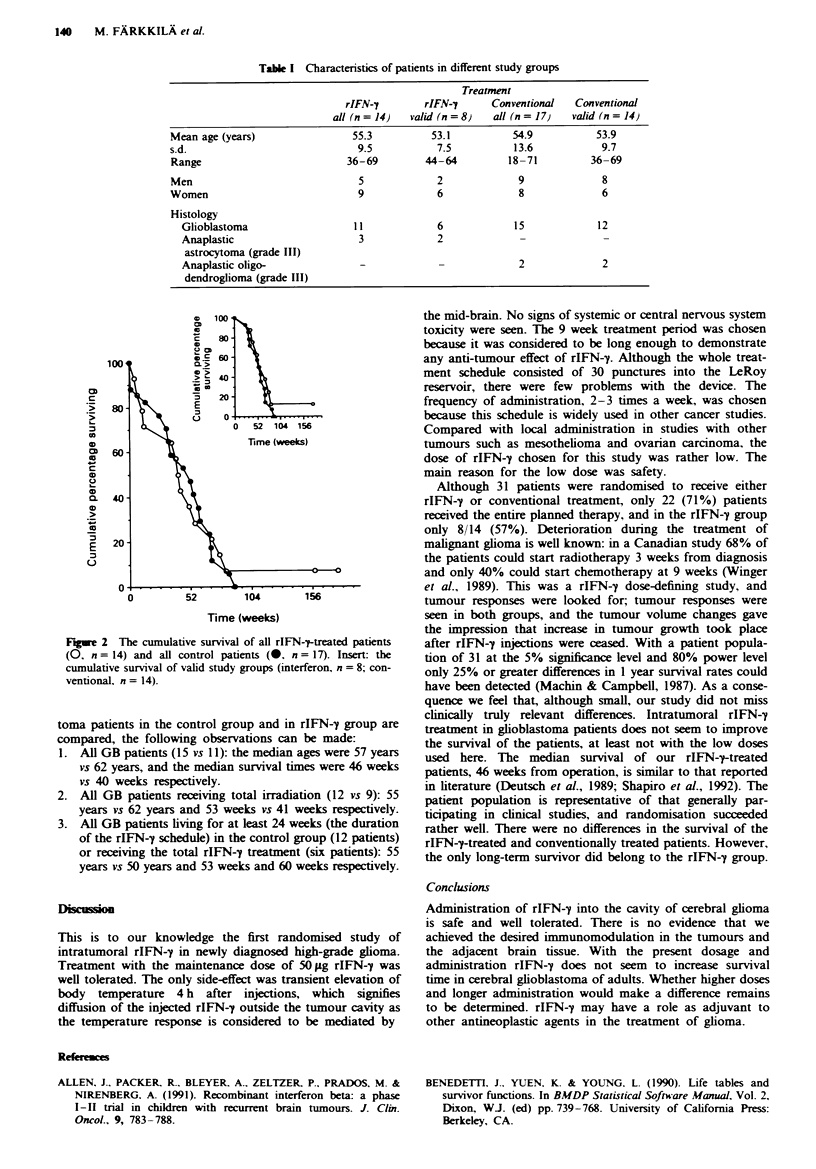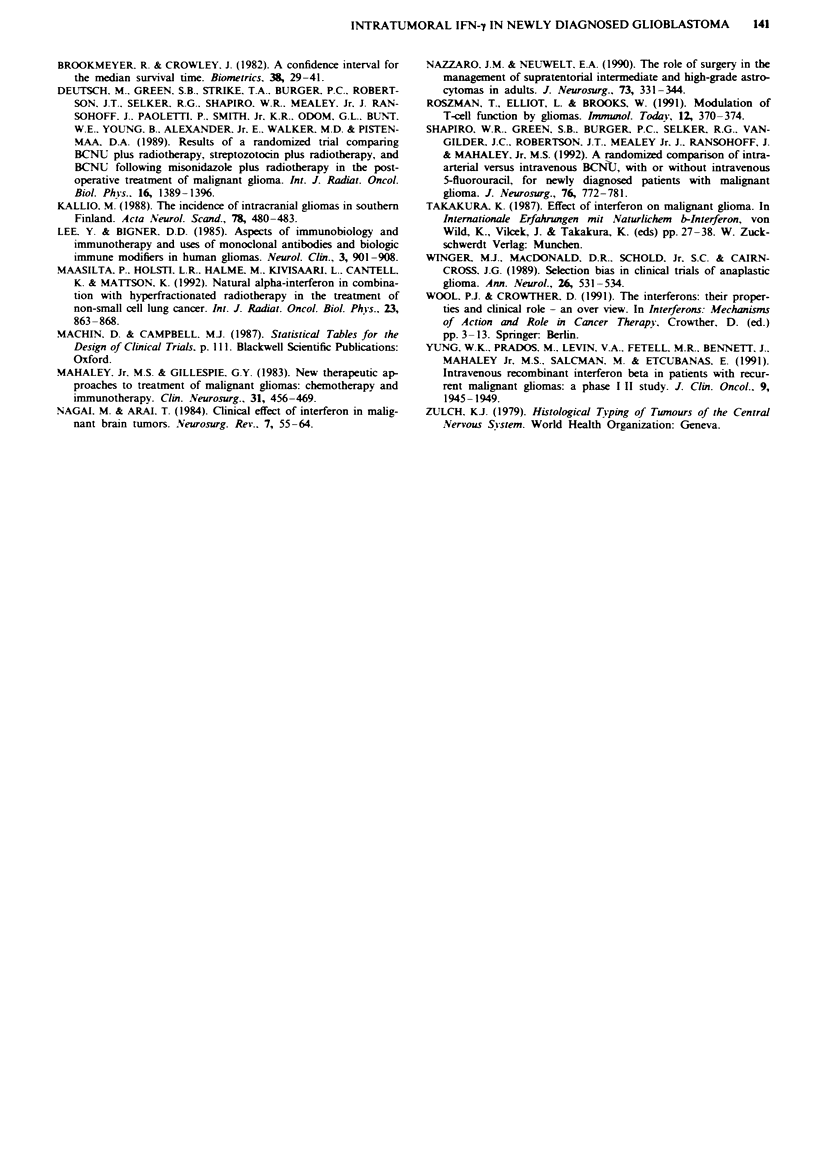# Randomised, controlled study of intratumoral recombinant gamma-interferon treatment in newly diagnosed glioblastoma.

**DOI:** 10.1038/bjc.1994.263

**Published:** 1994-07

**Authors:** M. Färkkilä, J. Jääskeläinen, M. Kallio, G. Blomstedt, R. Raininko, P. Virkkunen, A. Paetau, H. Sarelin, M. Mäntylä

**Affiliations:** Department of Neurology, University of Helsinki, Finland.

## Abstract

The effect of intratumoral recombinant interferon gamma (rIFN-gamma) as adjuvant to open cytoreduction and external irradiation of 60 Gy on survival in adults with a newly diagnosed high-grade cerebral glioma was studied. The patients were randomised during surgery into the rIFN-gamma group (n = 14) or the control group (n = 17), and the latter received a subcutaneous reservoir of rIFN-gamma injections. Intratumoral rIFN-gamma was given three times a week for 4 weeks until radiotherapy, escalating the dose from 5 micrograms to 50 micrograms. Both groups received external whole-brain irradiation of 40 Gy and a local boost of 20 Gy. After radiotherapy, rIFN-gamma was continued with 50 micrograms twice a week up to 9 weeks. The patients received no chemotherapy. Intratumoral rIFN-gamma was tolerated well with transient fever only. There were 12 glioblastomas (GBs) in the control group and nine in the rIFN-gamma group with completed irradiation. The patients were followed clinically and by computerised tomography (CT) every third month until death. Tumour responses were seen in three interferon-treated (one still alive 45 months after operation) and in two conventionally treated patients. The progression of the tumour volumes on CT did not differ between the IFN-treated and control groups. There were no differences in the survival times. Median survival of the rIFN-gamma-treated patients was 54 weeks (95% CI 35-68) and of the control patients 55 weeks (95% CI 41-77). Intratumoral rIFN-gamma given in the study doses does not seem to inhibit tumour growth or improve the prognosis of patients with high-grade glioma.


Br. J. Cancer (1994), 70, 138–141 © Macmillan Press Ltd., 1994

### Randomised, controlled study of intratumoral recombinant γ-interferon treatment in newly diagnosed glioblastoma

M. Färkkilä[1], J. Jääskeläinen[2], M. Kallio[1], G. Blomstedt[2], R. Raininko[3], P. Virkkunen[3], A. Paetau[5], H. Sarelin[6] & M. Mäntylä[4]

*Departments of [1]Neurology, [2]Neurosurgery, [3]Diagnostic Radiology, [4]Radiotherapy and Oncology and [5]Pathology, University of Helsinki, Helsinki, Finland; [6]Boehringer Ingelheim Inc., Espoo, Finland.*

**Summary** The effect of intratumoral recombinant interferon γ (rIFN-γ) as adjuvant to open cytoreduction and external irradiation of 60 Gy on survival in adults with a newly diagnosed high-grade cerebral glioma was studied. The patients were randomised during surgery into the rIFN-γ group ($n = 14$) or the control group ($n = 17$), and the latter received a subcutaneous reservoir of rIFN-γ injections. Intratumoral rIFN-γ was given three times a week for 4 weeks until radiotherapy, escalating the dose from 5 μg to 50 μg. Both groups received external whole-brain irradiation of 40 Gy and a local boost of 20 Gy. After radiotherapy, rIFN-γ was continued with 50 μg twice a week up to 9 weeks. The patients received no chemotherapy. Intratumoral rIFN-γ was tolerated well with transient fever only. There were 12 glioblastomas (GBs) in the control group and nine in the rIFN-γ group with completed irradiation. The patients were followed clinically and by computerised tomography (CT) every third month until death. Tumour responses were seen in three interferon-treated (one still alive 45 months after operation) and in two conventionally treated patients. The progression of the tumour volumes on CT did not differ between the IFN-treated and control groups. There were no differences in the survival times. Median survival of the rIFN-γ-treated patients was 54 weeks (95% CI 35–68) and of the control patients 55 weeks (95% CI 41–77). Intratumoral rIFN-γ given in the study doses does not seem to inhibit tumour growth or improve the prognosis of patients with high-grade glioma.

Glioblastomas (GBs) make up 35–75% of all primary brain tumours (Kallio, 1988). Glioblastoma multiforme and anaplastic astrocytoma are the most common and the most malignant. Malignant gliomas are treated surgically, usually with radiotherapy, and sometimes also with chemotherapy (Nazarro & Neuwelt, 1990), and these treatments prolong the life of a patient but practically never completely eradicate the tumour. Since the beginning of 1980s, interferons (IFNs) have been used to treat malignant gliomas. Mainly α (IFN-α) and β (IFN-β) have been tried (Nagai & Arai, 1982; Takakura, 1987; Allen *et al.*, 1991; Yung *et al.*, 1991). Promising results have also been reported with γ-interferon (IFN-γ) (Mahaley *et al.*, 1983).

Gliomas are immunosuppressive, and the defect is at least partially attributable to impaired T-cell function (Roszman *et al.*, 1991). The rationale of rIFN-γ treatment of glioma is to restore and enhance the local immune response against glioma tissue by up-regulation of glioma-associated and MHC antigens, and by recruitment and activation of leucocytes. IFN-γ may act antiproliferatively by unmasking antigenic determinants on the glioma cells and by inducing expression of receptors for tumour necrosis factor (TNF) on cells specific for IFN-γ (Woll & Crowther, 1991). rIFN-γ was chosen for clinical study because IFN-γ is the strongest inducer of Ia antigen, especially class II antigens (DRA antigens), expression and is also less neurotoxic than IFN-α or IFN-β, making high intracerebral doses possible. In addition, glioma patients may have reduced IFN-γ production as a result of reduced T-lymphocyte activity. This is of specific importance as IFN-γ is a potent activator of macrophages, and the production of IFN-γ is influenced by interleukin 2 (IL-2) (Lee & Bigner, 1985). In order to achieve sufficiently high concentrations of rIFN-γ in the tumour, rIFN-γ was administered locally into the tumour cavity.

In the present study rIFN-γ was administered locally into newly diagnosed malignant gliomas as adjuvant to open cytoreduction and external irradiation of 60 Gy. We chose to treat fresh tumours to exclude the confounding effects of previous surgery, radiotherapy and chemotherapy. Local injections of placebo in the control group were not ethically acceptable.

Correspondence: M. Färkkilä, Department of Neurology, University of Helsinki, Haartmanninkatu 4, SF-00290 Helsinki, Finland.
Received 6 December 1993; and in revised form 23 February 1994.

### Patients and methods

### *Objectives of the study*

1. To ascertain the safety of rIFN-γ administration into a cavity of cerebral glioma.
2. To study the effect of intratumoral rIFN-γ as adjuvant to open cytoreduction and external irradiation of 60 Gy on tumour control in adults with a newly diagnosed high-grade (III–IV) cerebral glioma.

### *Eligibility criteria*

1. Previously untreated high-grade (III–IV) cerebral glioma.
2. Age between 18 and 75 years.
3. Karnofsky performance scale over 60: needing at most occasional assistance.
4. No other previous or concurrent disease or serious condition likely to interfere with the treatment or assessment of the outcome.
5. Oral informed consent obtained. The study protocol was approved by the ethical committee of the Helsinki University Central Hospital.

### *Surgery and randomisation*

The patients were enrolled to the study between February 1988 and February 1991 from the patients referred to the Department of Neurosurgery, Helsinki University Central Hospital. Preliminary inclusion was based on CT. Randomisation took place during surgery by using numbered, sealed envelopes containing information on the treatment to be given. The supposed malignant glioma was first debulked from inside. If the frozen sections suggested a malignant glioma (grade III–IV), the patient was randomised before closure of the skull to the control group or the rIFN-γ group. Those of the rIFN-γ group received a subcutaneous LeRoy capsule with the tip of the catheter in the tumour cavity for local rIFN-γ administration. The final histological diagnosis was made by a neuropathologist (A.P.) from paraffin sections according to the WHO classification (Zülch, 1979).

*Treatment schedule*

The planned treatment schedule for the control group and for the rIFN-γ group is presented in Figure 1. After the resection all patients received 1 week's post-operative neurosurgical care. The injections of rIFN-γ into the LeRoy capsule were started on the seventh post-operative day, and rIFN-γ was administered three times a week for 4 weeks until radiotherapy, escalating the dose from 5 μg to 50 μg. rIFN-γ was not given during radiotherapy because of the possibility of enhancing radiation damage, as reported for natural IFN-α in lung cancer (Maasilta *et al.*, 1992). The planned radiotherapy was the same for all patients in both groups, starting in the fourth or fifth post-operative week. A total of 60 Gy was delivered in 30 fractions each of 2 Gy, five times a week, for either 6 or 9 weeks, depending on whether or not a 3 week pause was introduced. The first 20 fractions (40 Gy) were delivered to whole brain, followed by ten fractions (20 Gy) of local irradiation. The local irradiation was delivered to the target volume, planned to be the 2 cm margin outside the tumour border. After radiotherapy, rIFN-γ was continued with a dose of 50 μg twice a week for up to 9 weeks. The patients received no chemotherapy. Steroids, anticonvulsants and other medication were given according to clinical needs.

*Safety of rIFN-γ administration*

rIFN-γ was produced in *Escherichia coli* using gene technology by Boehringer Ingelheim. Symptoms and signs of possible side-effects were checked clinically, by ECG, by blood pressure and pulse rate (2 and 4 h after intratumoral rIFN-γ injections) and laboratory tests (complete blood analysis, thrombocytes, creatinine, aminotransferases, potassium, sodium, calcium, glucose, protein, serum and urine osmolality).

*Follow-up and imaging*

The tumours were imaged with CT before operation, before radiotherapy, after radiotherapy, and every third month until death. Magnetic resonance imaging (MRI) was performed without contrast enhancement as the contrast medium was not registered for general use in 1988. MRI was not used for tumour volume measurement; instead, tumour volumes were measured on contrast-enhanced CT scans by two radiologists who did not know the treatment the patient had received. The tumour volume was calculated by multiplying the three largest diameters, perpendicular to each other, and by using a spherical correction factor of π/6.

Tumour responses were considered complete if the volume of the tumour did not increase at all, and partial if the increase in volume was no greater than 25% in 6 months.

*Statistical analysis*

The cumulative survivals were computed using the product-limit method, and the difference between the survivals of the two groups was evaluated with the generalised Wilcoxon test using the BMDP statistical software (Benedetti, 1990). The confidence intervals (95% CI) for the median survivals were calculated according to Brookmeyer and Crowley (1982).

**Figure 1** The planned treatment schedule in the trial with rIFN-γ doses.

### Results

*Randomisation*

A total of 32 patients were randomised to receive open cytoreduction plus external irradiation of 60 Gy with or without intratumoral rIFN-γ. One patient in the rIFN-γ group was excluded because his tumour proved to be a metastatic adenocarcinoma: fresh-frozen sections had suggested a malignant glioma. The control group consisted of 17 patients and the rIFN-γ group consisted of 14 patients (Table I). The median ages were similar, 57 years (range 36–69) in the control group and 59 years (range 18–71) in the rIFN-γ group. The control group consisted of 15 patients with glioblastomas (GBs) and two with anaplastic oligodendrogliomas (AOs), and the rIFN-γ group consisted of 11 patients with GBs and three with anaplastic astrocytomas (AAs) (Table I).

*Valid study groups*

In the control group the whole treatment schedule took 14 weeks: two patients with GB died before radiotherapy and in one patient with GB radiotherapy was interrupted owing to poor clinical condition (Table I). In the rIFN-γ group the schedule took 24 weeks: two patients with GB died before radiotherapy and two patients with GB died before post-irradiation rIFN-γ therapy. In addition, one patient with GB did not receive the total post-irradiation rIFN-γ dose because his LeRoy capsule was removed because of suspicion of infection, which was not confirmed. One patient with AA obviously did not receive a total rIFN-γ dose because of subgaleal leakage of rIFN-γ during injection. The valid study groups thus included 14 control patients and eight rIFN-γ patients.

*rIFN-γ treatment*

Intratumoral rIFN-γ injections were given to 14 patients (Table I). The patients tolerated the scheduled dosage well, with only a slight increase in body temperature about 4 h after the injection. No signs of systemic or CNS toxicity were seen, and the blood parameters showed no noticeable changes. No change in pulse rate or blood pressure was seen.

*Tumour response to post-radiotherapy rIFN-γ*

After radiotherapy some tumour responses were seen. One rIFN-γ-treated patient is still alive 45 months after the operation with complete tumour response until a recently detected small recurrence (Figure 2). In two of eight patients receiving the whole planned dose of rIFN-γ the tumour volumes were reduced after the rIFN-γ treatment period, in one patient for 7 months and in the other one for 41 months. In the control group the volume remained stable in three patients. In the other patients the tumour grew relentlessly in spite of all treatments. In the valid rIFN-γ group ($n = 8$) there were two complete and one partial responses (3/8). In the valid conventional treatment group ($n = 14$), two complete responses were seen, giving response rates of 37.5% and 14.3%. The difference is not statistically significant.

*Survival analysis*

The median survival of all the patients was 46 weeks (95% CI 35–55), but one patient is still alive 45 months after operation. The median survivals of the rIFN-γ-treated and conventionally treated groups were 41 (95% CI 12–55) weeks and 52 (95% CI 31–60) weeks, respectively, for all patients (Figure 2), the difference being not statistically significant ($P = 91$). When analysing the survival of patients who received the whole planned treatments, the valid study groups, the median survival was 54 (95% CI 35–68) weeks for the conventional group and 55 (95% CI 41–77) weeks for the rIFN-γ group. The total rIFN-γ dose was 1.09–1.55 mg. There was no significant difference ($P = 0.35$) in the survival times of these groups either. When only the glioblas-

**Table I** Characteristics of patients in different study groups

| | *Treatment* | | | |
|---|---|---|---|---|
| | *rIFN-γ all (n = 14)* | *rIFN-γ valid (n = 8)* | *Conventional all (n = 17)* | *Conventional valid (n = 14)* |
| Mean age (years) | 55.3 | 53.1 | 54.9 | 53.9 |
| s.d. | 9.5 | 7.5 | 13.6 | 9.7 |
| Range | 36-69 | 44-64 | 18-71 | 36-69 |
| Men | 5 | 2 | 9 | 8 |
| Women | 9 | 6 | 8 | 6 |
| Histology | | | | |
| Glioblastoma | 11 | 6 | 15 | 12 |
| Anaplastic astrocytoma (grade III) | 3 | 2 | – | – |
| Anaplastic oligo-dendroglioma (grade III) | – | – | 2 | 2 |

**Figure 2** The cumulative survival of all rIFN-γ-treated patients (○, $n = 14$) and all control patients (●, $n = 17$). Insert: the cumulative survival of valid study groups (interferon, $n = 8$; conventional, $n = 14$).

toma patients in the control group and in rIFN-γ group are compared, the following observations can be made:

1. All GB patients (15 *vs* 11): the median ages were 57 years *vs* 62 years, and the median survival times were 46 weeks *vs* 40 weeks respectively.
2. All GB patients receiving total irradiation (12 *vs* 9): 55 years *vs* 62 years and 53 weeks *vs* 41 weeks respectively.
3. All GB patients living for at least 24 weeks (the duration of the rIFN-γ schedule) in the control group (12 patients) or receiving the total rIFN-γ treatment (six patients): 55 years *vs* 50 years and 53 weeks and 60 weeks respectively.

### Discussion

This is to our knowledge the first randomised study of intratumoral rIFN-γ in newly diagnosed high-grade glioma. Treatment with the maintenance dose of 50 μg rIFN-γ was well tolerated. The only side-effect was transient elevation of body temperature 4 h after injections, which signifies diffusion of the injected rIFN-γ outside the tumour cavity as the temperature response is considered to be mediated by the mid-brain. No signs of systemic or central nervous system toxicity were seen. The 9 week treatment period was chosen because it was considered to be long enough to demonstrate any anti-tumour effect of rIFN-γ. Although the whole treatment schedule consisted of 30 punctures into the LeRoy reservoir, there were few problems with the device. The frequency of administration, 2–3 times a week, was chosen because this schedule is widely used in other cancer studies. Compared with local administration in studies with other tumours such as mesothelioma and ovarian carcinoma, the dose of rIFN-γ chosen for this study was rather low. The main reason for the low dose was safety.

Although 31 patients were randomised to receive either rIFN-γ or conventional treatment, only 22 (71%) patients received the entire planned therapy, and in the rIFN-γ group only 8/14 (57%). Deterioration during the treatment of malignant glioma is well known: in a Canadian study 68% of the patients could start radiotherapy 3 weeks from diagnosis and only 40% could start chemotherapy at 9 weeks (Winger *et al.*, 1989). This was a rIFN-γ dose-defining study, and tumour responses were looked for; tumour responses were seen in both groups, and the tumour volume changes gave the impression that increase in tumour growth took place after rIFN-γ injections were ceased. With a patient population of 31 at the 5% significance level and 80% power level only 25% or greater differences in 1 year survival rates could have been detected (Machin & Campbell, 1987). As a consequence we feel that, although small, our study did not miss clinically truly relevant differences. Intratumoral rIFN-γ treatment in glioblastoma patients does not seem to improve the survival of the patients, at least not with the low doses used here. The median survival of our rIFN-γ-treated patients, 46 weeks from operation, is similar to that reported in literature (Deutsch *et al.*, 1989; Shapiro *et al.*, 1992). The patient population is representative of that generally participating in clinical studies, and randomisation succeeded rather well. There were no differences in the survival of the rIFN-γ-treated and conventionally treated patients. However, the only long-term survivor did belong to the rIFN-γ group.

### *Conclusions*

Administration of rIFN-γ into the cavity of cerebral glioma is safe and well tolerated. There is no evidence that we achieved the desired immunomodulation in the tumours and the adjacent brain tissue. With the present dosage and administration rIFN-γ does not seem to increase survival time in cerebral glioblastoma of adults. Whether higher doses and longer administration would make a difference remains to be determined. rIFN-γ may have a role as adjuvant to other antineoplastic agents in the treatment of glioma.

### References

ALLEN, J., PACKER, R., BLEYER, A., ZELTZER, P., PRADOS, M. & NIRENBERG, A. (1991). Recombinant interferon beta: a phase I–II trial in children with recurrent brain tumours. *J. Clin. Oncol.*, **9**, 783–788.

BENEDETTI, J., YUEN, K. & YOUNG, L. (1990). Life tables and survivor functions. In *BMDP Statistical Software Manual*, Vol. 2, Dixon, W.J. (ed) pp. 739–768. University of California Press: Berkeley, CA.

BROOKMEYER, R. & CROWLEY, J. (1982). A confidence interval for the median survival time. *Biometrics*, **38**, 29-41.

DEUTSCH, M., GREEN, S.B., STRIKE, T.A., BURGER, P.C., ROBERTSON, J.T., SELKER, R.G., SHAPIRO, W.R., MEALEY, Jr, J. RANSOHOFF, J., PAOLETTI, P., SMITH, Jr, K.R., ODOM, G.L., BUNT, W.E., YOUNG, B., ALEXANDER, Jr, E., WALKER, M.D. & PISTENMAA, D.A. (1989). Results of a randomized trial comparing BCNU plus radiotherapy, streptozotocin plus radiotherapy, and BCNU following misonidazole plus radiotherapy in the postoperative treatment of malignant glioma. *Int. J. Radiat. Oncol. Biol. Phys.*, **16**, 1389-1396.

KALLIO, M. (1988). The incidence of intracranial gliomas in southern Finland. *Acta Neurol. Scand.*, **78**, 480-483.

LEE, Y. & BIGNER, D.D. (1985). Aspects of immunobiology and immunotherapy and uses of monoclonal antibodies and biologic immune modifiers in human gliomas. *Neurol. Clin.*, **3**, 901-908.

MAASILTA, P., HOLSTI, L.R., HALME, M., KIVISAARI, L., CANTELL, K. & MATTSON, K. (1992). Natural alpha-interferon in combination with hyperfractionated radiotherapy in the treatment of non-small cell lung cancer. *Int. J. Radiat. Oncol. Biol. Phys.*, **23**, 863-868.

MACHIN, D. & CAMPBELL, M.J. (1987). *Statistical Tables for the Design of Clinical Trials*, p. 111. Blackwell Scientific Publications: Oxford.

MAHALEY, Jr, M.S. & GILLESPIE, G.Y. (1983). New therapeutic approaches to treatment of malignant gliomas: chemotherapy and immunotherapy. *Clin. Neurosurg.*, **31**, 456-469.

NAGAI, M. & ARAI, T. (1984). Clinical effect of interferon in malignant brain tumors. *Neurosurg. Rev.*, **7**, 55-64.

NAZZARO, J.M. & NEUWELT, E.A. (1990). The role of surgery in the management of supratentorial intermediate and high-grade astrocytomas in adults. *J. Neurosurg.*, **73**, 331-344.

ROSZMAN, T., ELLIOT, L. & BROOKS, W. (1991). Modulation of T-cell function by gliomas. *Immunol. Today*, **12**, 370-374.

SHAPIRO, W.R., GREEN, S.B., BURGER, P.C., SELKER, R.G., VANGILDER, J.C., ROBERTSON, J.T., MEALEY Jr, J., RANSOHOFF, J. & MAHALEY, Jr, M.S. (1992). A randomized comparison of intra-arterial versus intravenous BCNU, with or without intravenous 5-fluorouracil, for newly diagnosed patients with malignant glioma. *J. Neurosurg.*, **76**, 772-781.

TAKAKURA, K. (1987). Effect of interferon on malignant glioma. In *Internationale Erfahrungen mit Naturlichem b-Interferon*, von Wild, K., Vilcek, J. & Takakura, K. (eds) pp. 27-38. W. Zuckschwerdt Verlag: Munchen.

WINGER, M.J., MACDONALD, D.R., SCHOLD, Jr, S.C. & CAIRNCROSS, J.G. (1989). Selection bias in clinical trials of anaplastic glioma. *Ann. Neurol.*, **26**, 531-534.

WOOL, P.J. & CROWTHER, D. (1991). The interferons: their properties and clinical role - an over view. In *Interferons: Mechanisms of Action and Role in Cancer Therapy*, Crowther, D. (ed.) pp. 3-13. Springer: Berlin.

YUNG, W.K., PRADOS, M., LEVIN, V.A., FETELL, M.R., BENNETT, J., MAHALEY Jr, M.S., SALCMAN, M. & ETCUBANAS, E. (1991). Intravenous recombinant interferon beta in patients with recurrent malignant gliomas: a phase I/II study. *J. Clin. Oncol.*, **9**, 1945-1949.

ZULCH, K.J. (1979). *Histological Typing of Tumours of the Central Nervous System*. World Health Organization: Geneva.